# Self-Assembly of Angiotensin-Converting Enzyme Inhibitors Captopril and Lisinopril and Their Crystal Structures

**DOI:** 10.1021/acs.langmuir.1c01340

**Published:** 2021-07-22

**Authors:** Valeria Castelletto, Jani Seitsonen, Janne Ruokolainen, Sarah A. Barnett, Callum Sandu, Ian W. Hamley

**Affiliations:** † Department of Chemistry, University of Reading, Reading RG6 6AD, U.K.; ‡ Nanomicroscopy Center, Aalto University, Puumiehenkuja 2, Espoo FIN-02150, Finland; § Diamond Light Source, Harwell Science and Innovation Campus, Fermi Avenue, Didcot OX11 0DE, U.K.

## Abstract

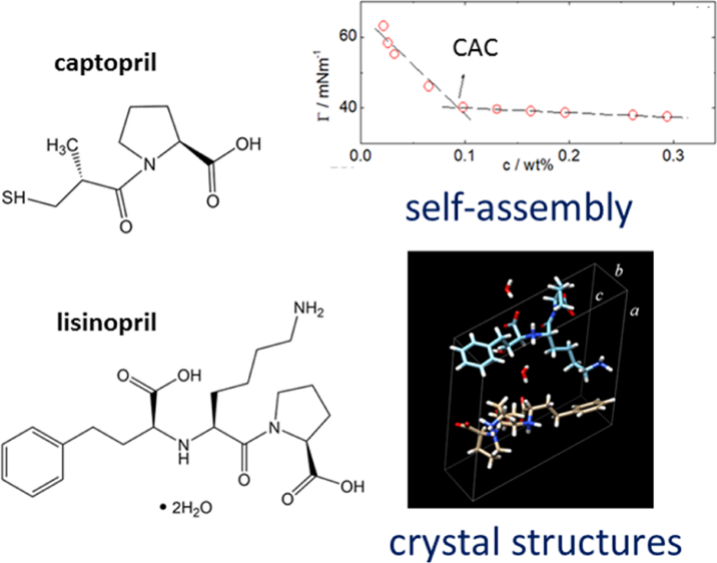

The peptide angiotensin-converting enzyme inhibitors captopril and lisinopril are unexpectedly shown to exhibit critical aggregation concentration (CAC) behavior through measurements of surface tension, electrical conductivity, and dye probe fluorescence. These three measurements provide similar values for the CAC, and there is also evidence from circular dichroism spectroscopy for a possible conformational change in the peptides at the same concentration. Cryogenic transmission electron microscopy indicates the formation of micelle-like aggregates above the CAC, which can thus be considered a critical micelle concentration, and the formation of aggregates with a hydrodynamic radius of ∼6–7 nm is also evidenced by dynamic light scattering. We also used synchrotron radiation X-ray diffraction to determine the single-crystal structure of captopril and lisinopril. Our results improve the accuracy of previous data reported in the literature, obtained using conventional X-ray sources. We also studied the structure of aqueous solutions containing captopril or lisinopril at high concentrations. The aggregation may be driven by intermolecular interactions between the proline moiety of captopril molecules or between the phenylalanine moiety of lisinopril molecules.

## Introduction

**Scheme 1:**
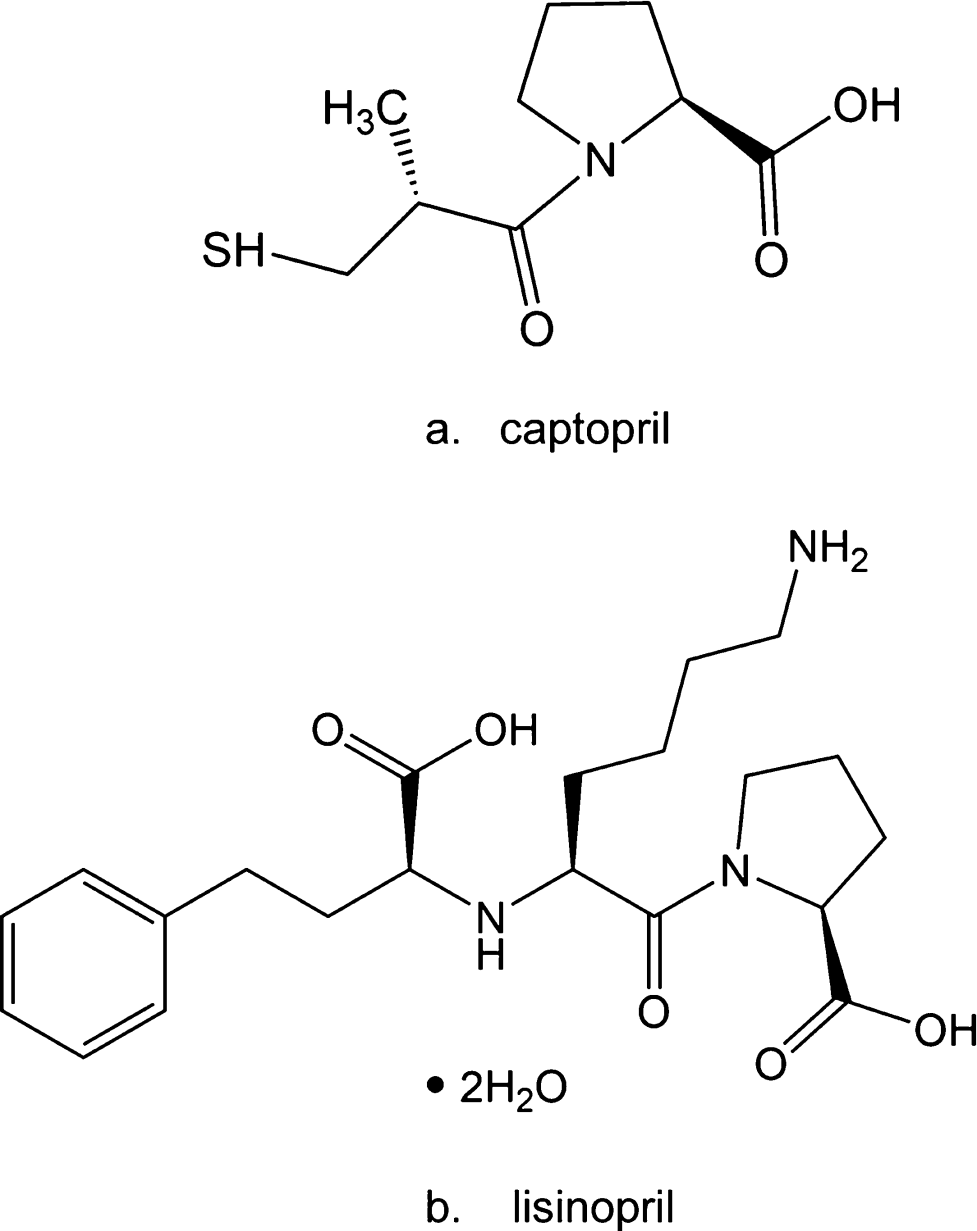
Chemical Structures for (a) Captopril and (b) Lisinopril

Angiotensin-converting inhibitors are a class of drugs mainly used for the treatment of high blood pressure and heart failure. High blood pressure can result from the overproduction of angiotensin by the angiotensin-converting enzyme (ACE).^1,2^ Captopril^3−5^ and lisinopril^6−8^ are drugs designed to inhibit ACE activity by binding to the active site of ACE, consequently inhibiting the conversion of angiotensin. Both captopril and lisinopril are peptide analogues since the captopril structure is inspired by the sequence of the Brazilian snake *Bothrops jararaca* venom peptide,^9^ while lisinopril is a synthetic modification of captopril. However, they differ in their functional binding group: captopril has a thiol-functional binding group, while lisinopril has a dicarboxyl-functional binding group (Scheme 1).^10,11^

Captopril and lisinopril are the active agents in many pharmaceutical formulations prescribed as ACE inhibitor drugs.^12^ Captopril is prescribed for the treatment of congestive heart failure and other high blood pressure-related diseases.^13,14^ Lisinopril shares similar active properties to captopril, which is used to treat several cardiac-related infections or malfunctions.^15−18^

Very recently, captopril and lisinopril were evaluated as antiviral drugs in the worldwide fight against the COVID-19 pandemic caused by the SARS-CoV-2 virus. Spike proteins on the surface of SARS-CoV-2 bind to ACE2 receptors, which the virus uses to get into human cells. Since the proposed mechanism of activity of captopril and lisinopril involves interference in the production of angiotensin 2 or blocking ACE2, clinical trials to test captopril or lisinopril use in treating COVID-19 are underway.^19^ Another recently described application demonstrates the ability of captopril to inhibit amyloid fibril formation.^20^ Many other peptides are being researched as ACE2 inhibitors due to the high level of current interest in potential COVID-19 treatments.^21−23^

Due to their potential activity as antiviral drugs and since captopril and lisinopril are active in a fluid environment, it is valuable to study their behavior in solution. Here, we study for the first time the aggregation of captopril and lisinopril in aqueous solution. This is motivated by the observation that the molecules may show self-assembly behavior, although this has not been explored, to the best of our knowledge. In addition, we performed synchrotron radiation X-ray diffraction (SR-XRD) to confirm previously reported single-crystal structures.^24−28^ A powerful combination of fluorescence, circular dichroism (CD), and cryogenic-transmission electron microscopy (Cryo-TEM) methods was used to probe the self-assembly of captopril and lisinopril in solution. Cryo-TEM reveals the presence of micellar structures that form above critical aggregation concentrations (CACs) determined from fluorescence probe and CD spectroscopy measurements.

## Experimental Section

### Materials

Captopril and lisinopril (Mw_captopril_ = 217.29 g/mol and Mw_lisinopril_ = 441.52 g/mol; the chemical structure is displayed in Scheme 1) were purchased from Sigma-Aldrich (UK) and used as received. Samples for solution characterization were prepared by dissolving weighed amounts of captopril or lisinopril with weighed amounts of ultrapure water (18 MΩ ThermoFisher Barnstead). Samples for crystallization were prepared by mixing weighed amounts of captopril or lisinopril with measured volumes of diethyl ether. The pH measurements were performed with a Mettler Toledo FiveEasy pH meter with a Sigma-Aldrich micro-pH combination electrode (glass body).

### Mass Spectroscopy

All reagents were of LCMS grade. Samples were diluted to a final concentration of 30 μg/mL in water. 5 μL of the sample was injected into a Thermo Scientific Accela HPLC. The LC buffers were (A) water and (B) acetonitrile, both with 0.1% formic acid. The column used for high-performance liquid chromatography (HPLC) was a Thermo Hypersil Gold 2.1 × 50 mm C18 column, with a particle size of 1.9 μm and a pore size of 175 Å.

The mass spectrometer used was a Thermo Scientific LTQ-Orbitrap-XL. MS1 scans were performed on the Orbitrap at 100 K resolution in positive ion mode scanning across the 85–2000 *m*/*z* range. A lock mass was used (413.266230 g mol^–1^). Our mass spectra (shown in Supporting Information, Figure S1) confirm the high purity of the purchased samples, with peaks corresponding to the expected products and no evidence for contaminants.

### Fluorescence Spectroscopy

The fluorescence probe 8-anilo-1-naphthalenesulfonic acid (ANS) was used to locate the CAC. The ANS fluorophore is sensitive to the hydrophobicity of its surrounding environment,^29^ making it suitable to determine a CAC value resulting from hydrophobic collapse. Samples for ANS were prepared by the dilution of a mother sample prepared as 1.6 wt % captopril or 1.4 wt % lisinopril in 2 × 10^–3^ wt % ANS. Spectra were recorded with a Varian Cary Eclipse fluorescence spectrometer with samples in 4 mm inner width quartz cuvettes. ANS assays were performed by measuring spectra from 400 to 670 nm (λ_ex_ = 356 nm). Results from the ANS assays were analyzed as *I*/*I*_0_*versus* concentration *c*, where *I* is the maximum intensity of emission for solutions with captopril or lisinopril, while *I*_0_ is the maximum emission intensity for the ANS solution with no additives.

### Surface Tension

The critical micellar concentration (CMC) was determined by measuring the static surface tension Γ as a function of the concentration of the solution using the Du Noüy ring method. An automatic processor tensiometer (Krüss, model K12) which provides a temperature-controlled (±0.1 °C) environment for the sample was used to measure Γ at 20 °C. A series of solutions of captopril or lisinopril in doubly distilled deionized water were obtained by the sequential dilution of a concentrated stock solution. An equilibration period of 15 min was allowed before the measurement of Γ for each concentration.

### Electrical Conductivity

The conductivity κ was measured for the solutions of captopril and lisinopril using a Hanna Primo5 conductivity meter calibrated with a 1413 μS cm^–1^ calibration solution. The measurements were recorded three times for each concentration (the mean value is presented), starting from the most dilute to the most concentrated solution.

### CD Spectroscopy

CD spectra were recorded using a Chirascan spectropolarimeter (Applied Photophysics, UK). Solutions were placed between parallel plates (0.01 mm path length). Spectra were measured with a 0.5 nm step, 1 nm bandwidth, and 1 s collection time per step. The CD signal from the water background was subtracted from the CD data of the sample solutions. The sample dilution series was started from a 1.6 wt % captopril or from a 1.7 wt % lisinopril mother solution. The range of concentrations studied by CD includes the range of concentrations studied for the ANS assay using fluorescence spectroscopy.

### Fourier-Transform Infrared Spectroscopy

Spectra were recorded using a Thermo Scientific Nicolet iS5 instrument equipped with a DTGS detector, with a Specac Pearl liquid cell (sample contained between fixed CaF_2_ plates). Spectra (1 wt % sample in H_2_O) were scanned 128 times over the range of 900–4000 cm^–1^.

### Dynamic Light Scattering

Experiments were performed using an ALV CGS-3 system with a 5003 multidigital correlator. The light source was a 20 mW He-Ne laser, linearly polarized, with λ = 633 nm. A fixed scattering angle θ = 90° was used for all the experiments. Solutions were filtered through 0.20 μm Anotop filters from Whatman into standard 0.5 cm diameter cylindrical glass cells. Dynamic light scattering (DLS) experiments measured the intensity correlation function *g*(θ, *t*), where *t* is the lag time. A cumulant analysis algorithm built in the ALV CGS-3 system acquisition software was used to calculate the size-weighted distribution function from *g*(θ, *t*).

### Cryogenic Transmission Electron Microscopy

Experiments were performed using a field-emission cryo-electron microscope (JEOL JEM3200FSC), operating at 200 kV and at −187 °C, configured in the bright field mode and zero-loss energy filtering (omega type) with a slit width of 20 eV. The sizes of selected nanoassemblies were measured from photographs recorded using a Gatan Ultrascan 4000 CCD camera. Vitrified specimens were prepared on QUANTIFOIL 3.5/1 holey carbon copper grids (hole size of 3.5 μm). The grids were first plasma cleaned using a Gatan Solarus 9500 plasma cleaner and then transferred into the environmental chamber of an automated FEI Vitrobot device at room temperature and 100% humidity. Thereafter, 3 μL of the sample solution was applied on the grid, which was blotted twice for 5 s and then vitrified in a 1:1 mixture of liquid ethane and propane at a temperature of −180 °C. The grids with the vitrified sample solution were maintained at liquid nitrogen temperature and then cryo-transferred to the microscope.

### Crystal Preparation

Crystals were obtained by evaporating aliquots of 7 wt % captopril or 0.2 wt % lisinopril dissolved in diethyl ether on a microscopic slide placed inside a Petri dish. A second Petri dish was placed on top, and a small aperture was allowed between the Petri dishes to allow for the slow evaporation of diethyl ether over a period of 1 week. Thereafter, the aperture between the Petri dishes was closed. The crystals were stored on microscopic slides contained inside the Petri dishes sealed with parafilm until SR-XRD experiments.

### Synchrotron Radiation X-ray Diffraction

Measurements were performed on experiment hutch 1 (EH1) of Beamline I19 at the Diamond Light Source.^30^ The data were collected at a wavelength of 0.6889 Å on a Fluid Film Devices 3-circle fixed-chi diffractometer using a Dectris Pilatus 2 M detector. The crystal was mounted on a MiTeGen micromount using perfluoropolyether oil and cooled for data collection by a cryostream nitrogen-gas stream.^31^ The collected frames were integrated using DIALS,^32^ as implemented by xia2,^33^ and the data were corrected for absorption effects using a DIALS scale,^34^ an empirical method. The structure was solved by dual-space methods^35^ and refined by least-squares refinement on all unique measured *F*^2^ values.^36^

## Results and Discussion

We first determined the crystal structure of the two molecules, which also provides a quality control of the compounds studied in this work. Previous works report the crystal structure of captopril^24−26^ or lisinopril^27,28^ determined using conventional X-ray sources. Here, by using SR-XRD, we examine the accuracy and improve the resolution of the crystal structure already reported.

**Figure 1:**
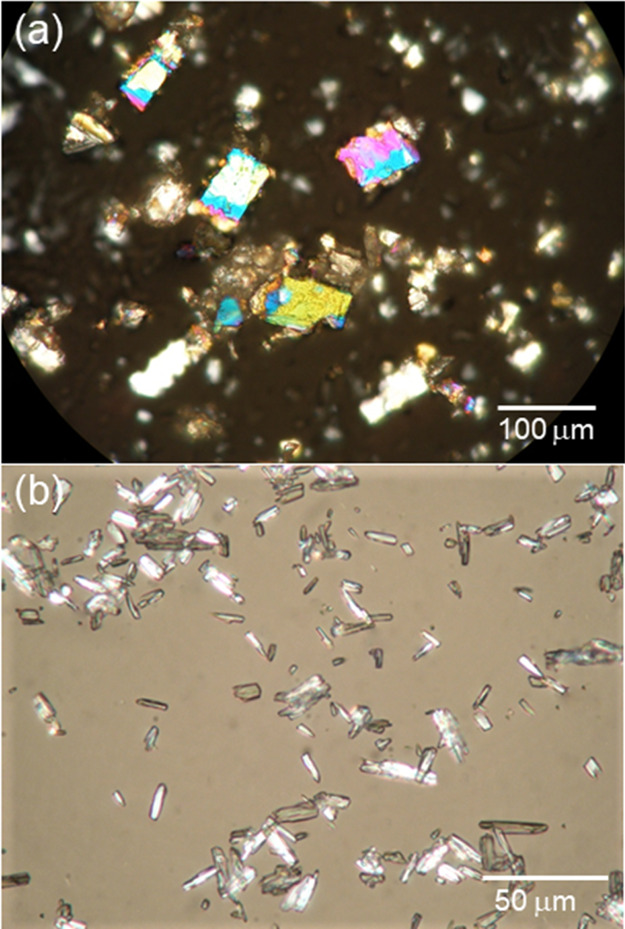
Representative images for crystals obtained by evaporating drops of (a) 7 wt % captopril in diethyl ether or (b) 0.2 wt % lisinopril in diethyl ether on a microscope slide.

A number of methods have been used before to obtain captopril or lisinopril crystals. In a previous work, captopril crystals have been grown by the vapor-diffusion technique using ethyl acetate as the solvent and petroleum ether as the precipitant.^24^ Captopril crystals were also obtained by slow evaporation from acetone or tetrahydrofuran–water solutions.^26^ Lisinopril crystals were obtained by the vapor-diffusion technique using water as a solvent and acetonitrile as the precipitant.^27^ Other groups explored the controlled dehydration of lisinopril crystals by recrystallizing the material from water solutions.^28^ Here, both captopril and lisinopril crystals were obtained by the evaporation of solutions of these materials in diethyl ether. Lisinopril crystals had a tendency to dry in compact crystalline aggregate structures. Therefore, the solutions of lisinopril used for crystallization were relatively diluted to avoid the formation of uniform plaques of crystals on the dried surfaces. Figure 1a,b shows the photographs of the crystals obtained by the evaporation of solutions containing 7 wt % captopril in diethyl ether (Figure 1a) or 0.2 wt % lisinopril in diethyl ether (Figure 1b). Square crystals of captopril similar to those displayed in Figure 1a, already reported in the literature, are the alternative to plate-like crystals of captopril associated with the disulfide bridged captopril dimer.^26^ Lisinopril has been reported to crystallize in flexible-like needles,^27^ in contrast to the rectangular crystals in Figure 1b.

**Figure 2:**
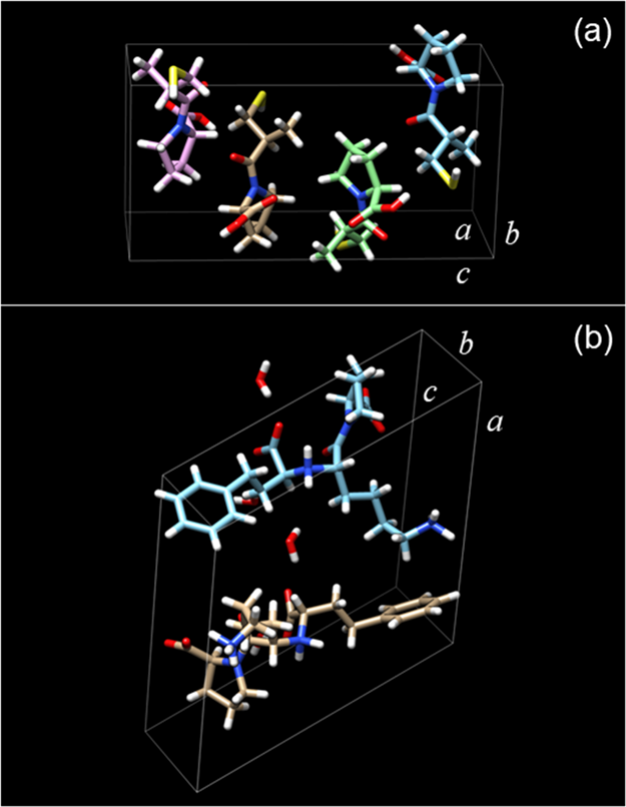
Unit cells for (a) captopril and (b) lisinopril calculated using the parameters in Table 1.

**Figure 3:**
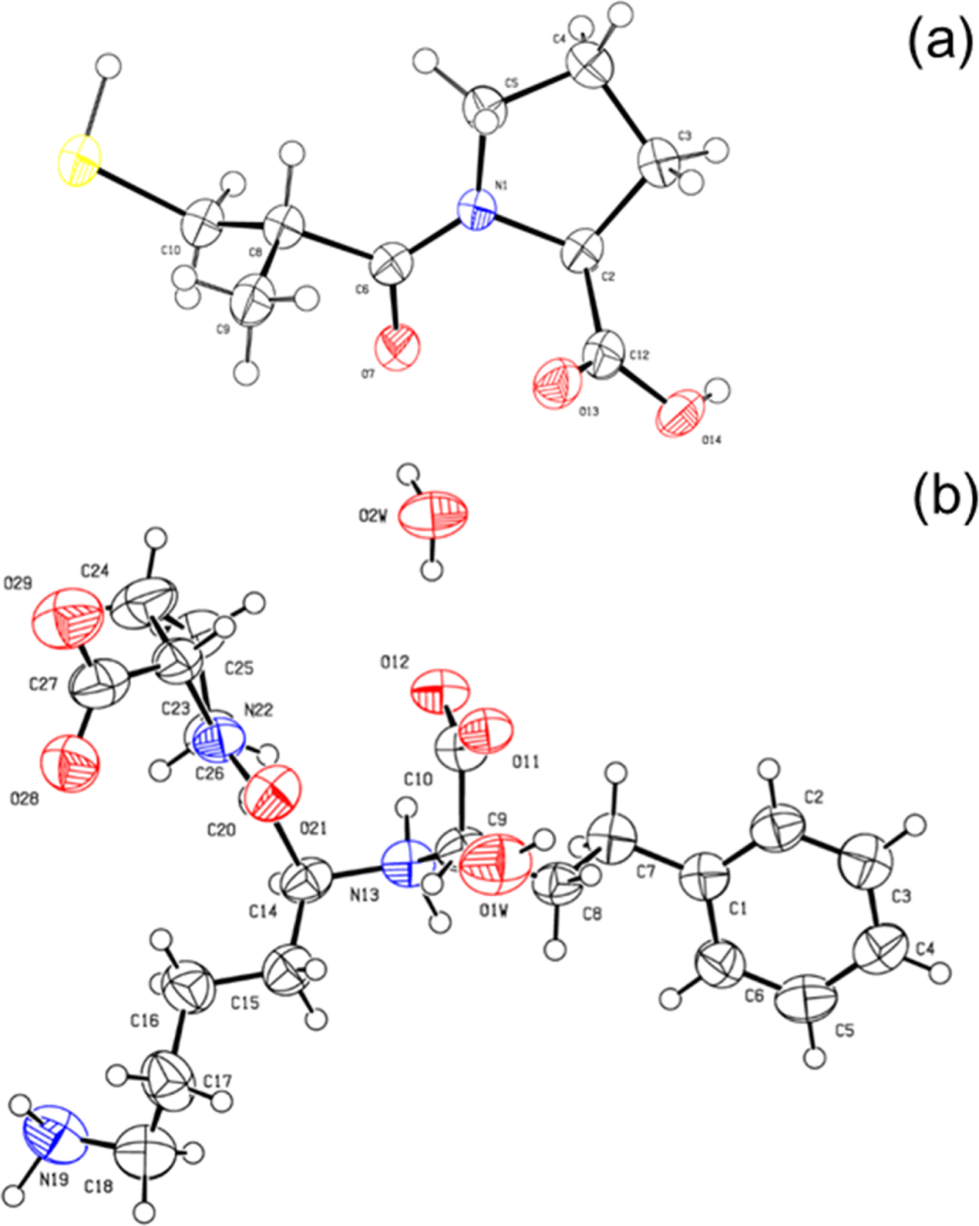
Structure and atomic numbering of the asymmetric unit in (a) captopril and (b) lisinopril crystals. Thermal ellipsoids are drawn at the 50% probability level.

The crystals shown in Figure 1a,b were sufficiently large to enable synchrotron single-crystal XRD structure determination. Crystal structure determination showed that captopril crystallizes in an orthorhombic *P*2_1_2_1_2_1_ structure, with unit cell parameters (Table 1 and Figure 2a) very similar to those reported in the literature for crystals grown by the vapor-diffusion technique using ethyl acetate as the solvent and petroleum ether as the precipitant.^24^ There was no evidence for captopril dimerization (due to disulfide bond formation)^37−40^ in the obtained crystal structure or diffraction data. The lisinopril crystal structure is sensitive to the humidity of the sample. Powder XRD was used to prove that lisinopril can be crystallized in its monohydrate, dihydrate, or anhydrous forms depending on crystal hydration.^28^ Here, lisinopril crystallizes in a monoclinic *P*2_1_ structure, with unit cell parameters (Table 1 and Figure 2b) very similar to those reported in the literature for single-crystal lisinopril dehydrate.^28^Figure 3 shows the atom numbering corresponding to the unit cell structures shown in Figure 2.

**Table 1:** Crystallographic Data for Captopril and Lisinopril

	captopril	lisinopril
bond precision/Å	C–C = 0.0067	C–C = 0.0086
wavelength/Å	0.68890	0.68890
chemical formula	C_9_H_15_NO_3_S	C_21_H_31_N_3_O_5_·2H_2_O
formula weight	217.28	441.52
crystal system	orthorhombic	monoclinic
space group	*P*2_1_2_1_2_1_	*P*2_1_
*Z*	4	2
*a*/Å	6.7844(2)	14.2303(13)
*b*/Å	8.7679(3)	5.8934(5)
*c*/Å	17.5411(8)	14.5405(15)
α/°	90	90
β/°	90	112.973(9)
γ/°	90	90
*V*/Å^3^	1043.43(7)	1122.7(2)
ρ_calc_/g cm^–1^	1.383	1.306
crystal dimensions/mm	0.100, 0.045, 0.020	0.040, 0.010, 0.005
*T*/K	100(2)	100(2)
μ/mm^–1^	0.227	0.06
reflexions		
F000	464.0	476.00
F000′	464.66	476.22
*h*,*k*,*l*_max_	8,10,20	16,7,17
unique reflections/*R*_int_	1838/0.0784	3733/0.1084
abscorr type, *T*_min_, *T*_max_	empirical, 0.997, 1.000	empirical, 0.999, 1.000
diffrn_measured_fraction_theta_full	99.5	99.0
theta (max)	24.212	24.274
*R*_1_ [*F*^2^ > 2σ], *wR*_2_ [all data]	0.0486, 0.1050	0.0492, 0.1087
goodness of fit (S)	0.935	0.796
parameters	137	316

Having determined the crystal structure of the compounds, we examined potential aggregation and self-assembly in aqueous solution. A powerful combination of methods sensitive to colligative properties and the formation of hydrophobic domains was used, that is, surface tensiometry, electrical conductivity, and dye probe fluorescence measurements.^41−48^

**Figure 4:**
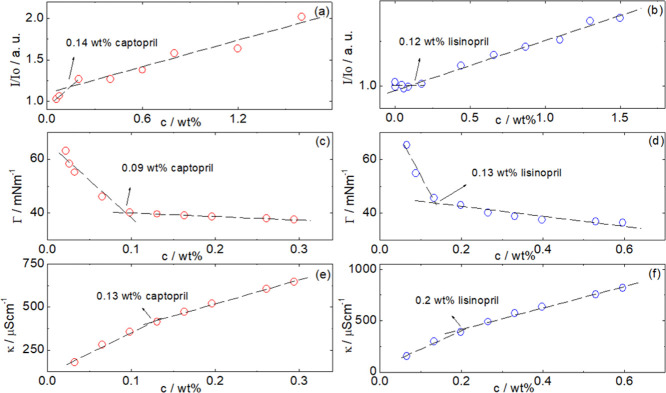
CAC measured by (a,b) ANS fluorescence, (c,d) surface tension, and (e,f) conductivity for captopril and lisinopril solutions.

Fluorescence measurements performed using ANS were undertaken to identify any CAC of captopril or lisinopril in water. Figure 4a,b shows the dependence of the normalized maximum intensity *I*/*I*_0_ as a function of the concentration. Figure 4a,b shows that the intensity of the ANS fluorescence emission grows, while the emission wavelength decreases with the increasing additive concentration. This result is characteristic of hydrophobic pocket formation with the increasing concentration of captopril or lisinopril. CAC values can be determined from the discontinuity in the dependence of *I*/*I*_0_ with the concentration, as indicated for captopril (Figure 4a) and lisinopril (Figure 4b).

The concentration dependence of surface tension is shown for captopril and lisinopril in Figure 4c,d, respectively. Discontinuities in the concentration dependence and leveling out of the surface tension at higher concentration are characteristic of CMC behaviour,^44,49,50^ and the values indicated in the figure are in very good agreement with those obtained from the fluorescence probe measurements (Figure 4a,b).

Electrical conductivity is another colligative property measurement sensitive to molecular aggregation which influences the number of ions present in solution. In the case of captopril and lisinopril, ionization is associated with the C-terminal carboxylate for both molecules and the lysine ε-amine group for lisinopril. The pH of solutions of the two peptides was measured as a function of concentration (Supporting Information, Figure S2). The pH at concentrations above the CAC for captopril is below pH 3. This is close to the expected p*K*_a_ of the carboxyl terminus^18^ ensuring a significant proportion of charged carboxyl groups. For lisinopril, the pH plateaus at pH 5.4, which is substantially below the expected p*K*_a_ = 10.5 for the lysine residue,^18^ so this residue will be charged as well as the carboxyl terminus. A discontinuity in electrical conductivity against concentration is also often used to detect micellization.^44,50^ Remarkably, both captopril and lisinopril show discontinuities in conductivity as a function of concentration, as shown in Figure 4e,f, respectively. The indicated CAC/CMC values are in excellent agreement with those obtained from the other two independent methods.

Our data from three independent measurement techniques thus indicate that both captopril and lisinopril aggregate in aqueous solution above ca. 0.1–0.2 wt %, specifically (5.5 ± 1.4) mM for captopril and (3.4 ± 1.0) mM for lisinopril. We emphasize the fact that both compounds crystallize into well-defined structures consistent with prior data, which is consistent with high-purity samples. This was also confirmed by our own mass spectrometry analysis (Supporting Information, Figure S1), so changes in colligative property data with concentration are not consistent with the presence of impurities. Fourier-transform infrared spectroscopy (FTIR) spectra were measured to check for the presence of possible dimers of captopril which can form in aqueous solution.^37−40^ The spectrum shown in Supporting Information, Figure S3 shows the presence of a peak at 2566 cm^–1^, which is assigned to the S–H stretching mode of free captopril molecules.^40^ This indicates that in the solutions studied, captopril is present mainly in the undimerized form.

**Figure 5:**
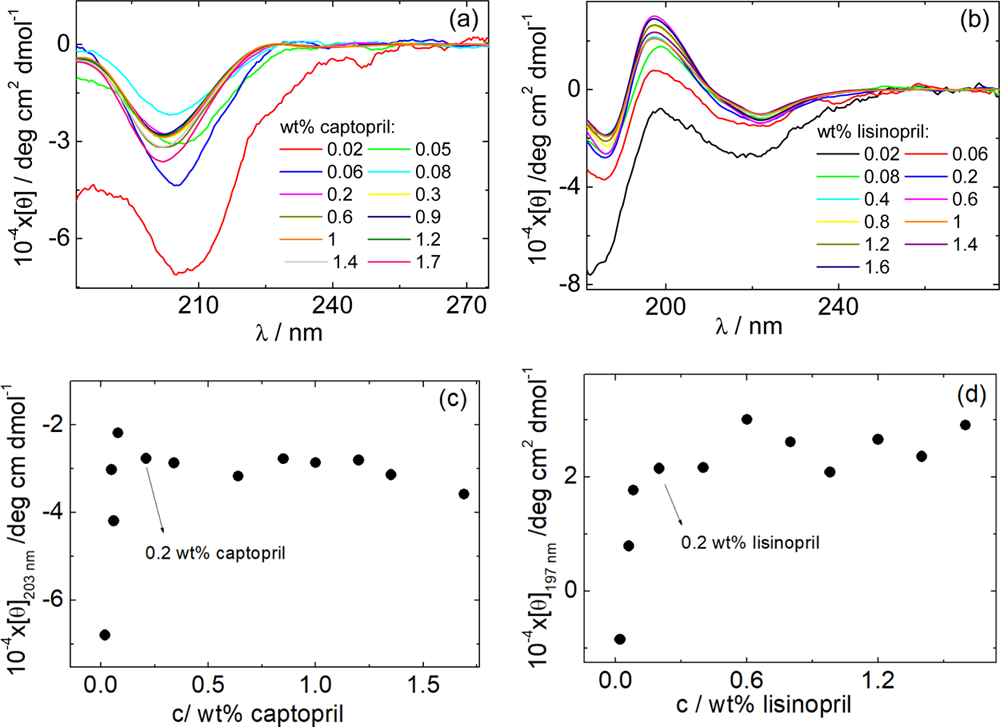
CD spectra as a function of the concentration for solutions containing (a) captopril and (b) lisinopril. (c) Concentration dependence of [θ]_203_ determined from the spectra in (a). (d) Concentration dependence of [θ]_197_ [from (b)].

Figure 5a,b shows the dependence of the CD signal measured for the same range of captopril or lisinopril concentrations as in Figure 4. The CD spectra provide conformational information on the peptide structures due to the amide backbone and the side groups.^51,52^

The CD spectra for captopril are characterized by a pronounced minimum at 203 nm (Figure 5a). The CD data reported in the literature for 1.3 × 10^–3^ wt % captopril^53^ and for 1.8 × 10^–3^ wt % captopril^54^ in water display a negative peak at 210 or 208 nm, close to 203 nm reported in Figure 5a, which was ascribed to the contributions from the n → π* transition of the carboxylate group and from the n → σ* transition of the amino group of the proline moiety (Scheme 1a).^54−56^ Peaks in this range are also affected by backbone amide π–π* and n−π* transitions.^56,57^ The CD spectra indicate that captopril does not form any organized secondary structure under the conditions studied. This is in contrast to some of the amino acids discussed in ref (55), which form fibrils at sufficiently high concentration, for example, phenylalanine^58^ or tryptophan.^59^Figure 5a also reveals a slight red shift in the position of the minimum upon reducing the concentration from 203 nm at high concentration to 205–208 nm for the lowest concentrations. This is consistent with the observation in ref (53), which reports a red shift on increasing pH (in our measurements, reducing concentration corresponds to higher pH; Supporting Information, Figure S2). In the case of ref (53), however, pH was varied at a fixed concentration.

The CD spectra for lisinopril are characterized by two minima at 186 and 222 nm and one maximum at 197 nm (Figure 5b). To the best of our knowledge, CD data have not previously been reported for lisinopril solutions. The positive peak in the CD spectra at 197 nm may be influenced by π–π stacking interactions due to the stacking of the aromatic ring in the phenylalanine moiety (which typically gives a peak at 210–230 nm) (Scheme 1b).^55,60,61^ Phe–Phe excitonic coupling contributions can significantly influence the peak position,^62,63^ as can such contributions from proline.^55^ The minima at 186 and 222 nm are influenced by the aforementioned amide backbone electronic transitions as well as the n → π* transition of the carboxylate group and the n → σ* transition of the amino group of the proline moiety (Scheme 1b).^51,54,55^ In contrast to the case of captopril, there is no evidence for a concentration-dependent red shift in the CD spectra for lisinopril in Figure 5b.

The data in Figure 5a,b were used to generate the plots displayed in Figure 5c,d. Figure 5c shows the dependence of the ellipticity at 203 nm, [θ]_203_, with concentration of captopril, while Figure 5d shows the dependence of [θ]_197_ with the concentration of lisinopril.

The data in Figure 5c,d follow similar patterns. Both [θ]_203_ and [θ]_197_ increase sharply with concentration until they reach a constant value at *c*_captopril_ = *c*_lisinopril_ – 0.2 wt %. This is assigned as the CAC for these molecules. Intermolecular interactions between proline moieties of captopril or between aromatic units in the phenylalanine moiety of lisinopril are saturated at concentrations higher than 0.2 wt % and therefore become independent of concentration. This process is correlated with the cooperative formation of hydrophobic pockets with increasing concentration, as revealed by the ANS fluorescence results shown in Figure 4a,b.

**Figure 6:**
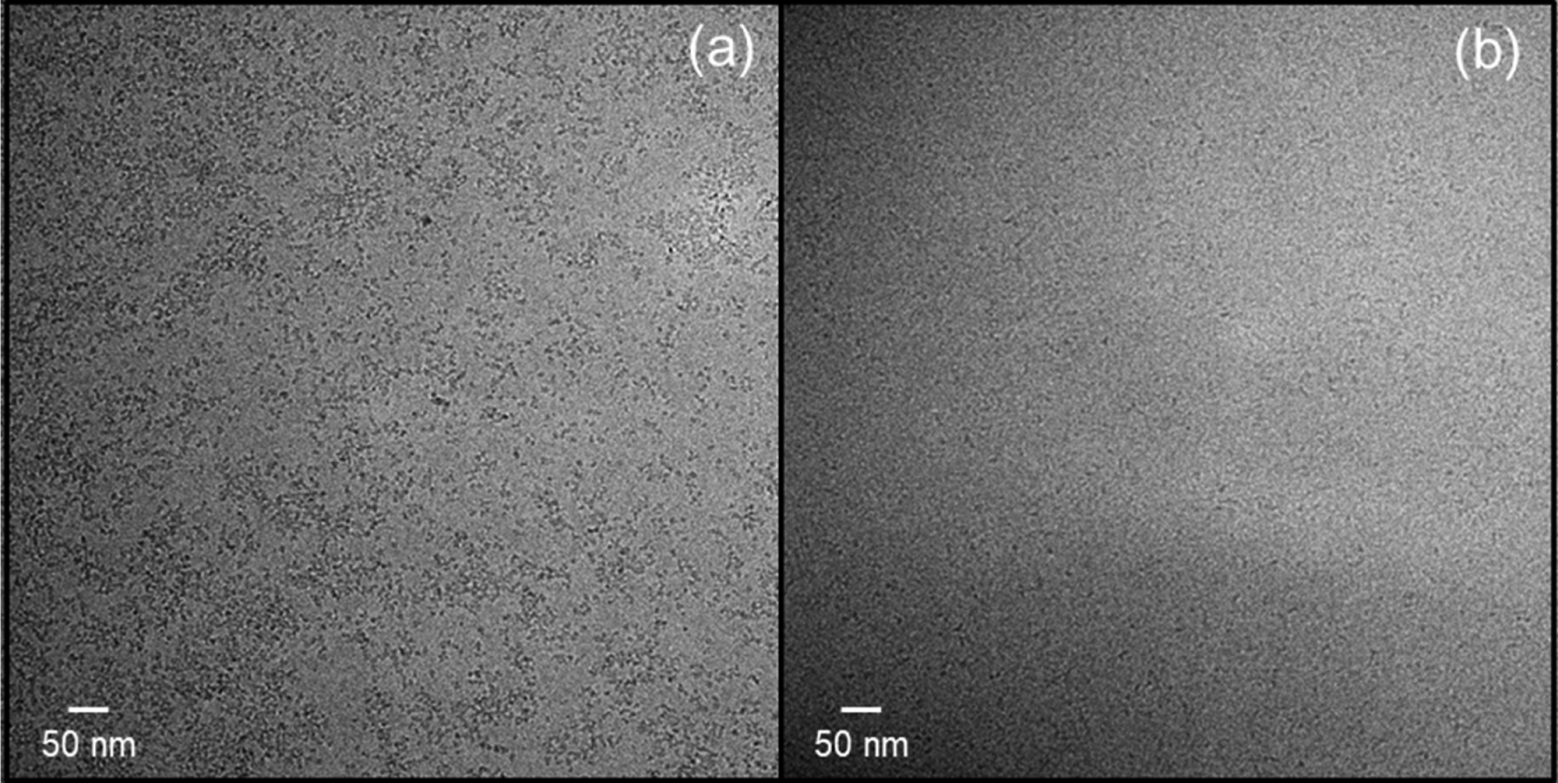
Cryo-TEM images for 1 wt % aqueous solutions of (a) captopril and (b) lisinopril.

Cryo-TEM is a powerful method to image nanostructures in self-assembled materials based on the vitrification of aqueous solutions, avoiding artefacts due to sample drying in the preparation of samples for conventional TEM or atomic force microscopy. Cryo-TEM was used to image the self-assembled nanostructures at a concentration well above the CAC of captopril and lisinopril. Representative cryo-TEM images are shown in Figure 6a,b for 1 wt % solutions of captopril and lisinopril, respectively. Both cryo-TEM images reveal the formation of very small aggregates in solution, <10 nm in diameter. These appear to be spherical micelle-like structures. These are not present in control images obtained for 0.05 wt % aqueous solutions (below the CAC) shown in Supporting Information, Figure S4.

**Figure 7:**
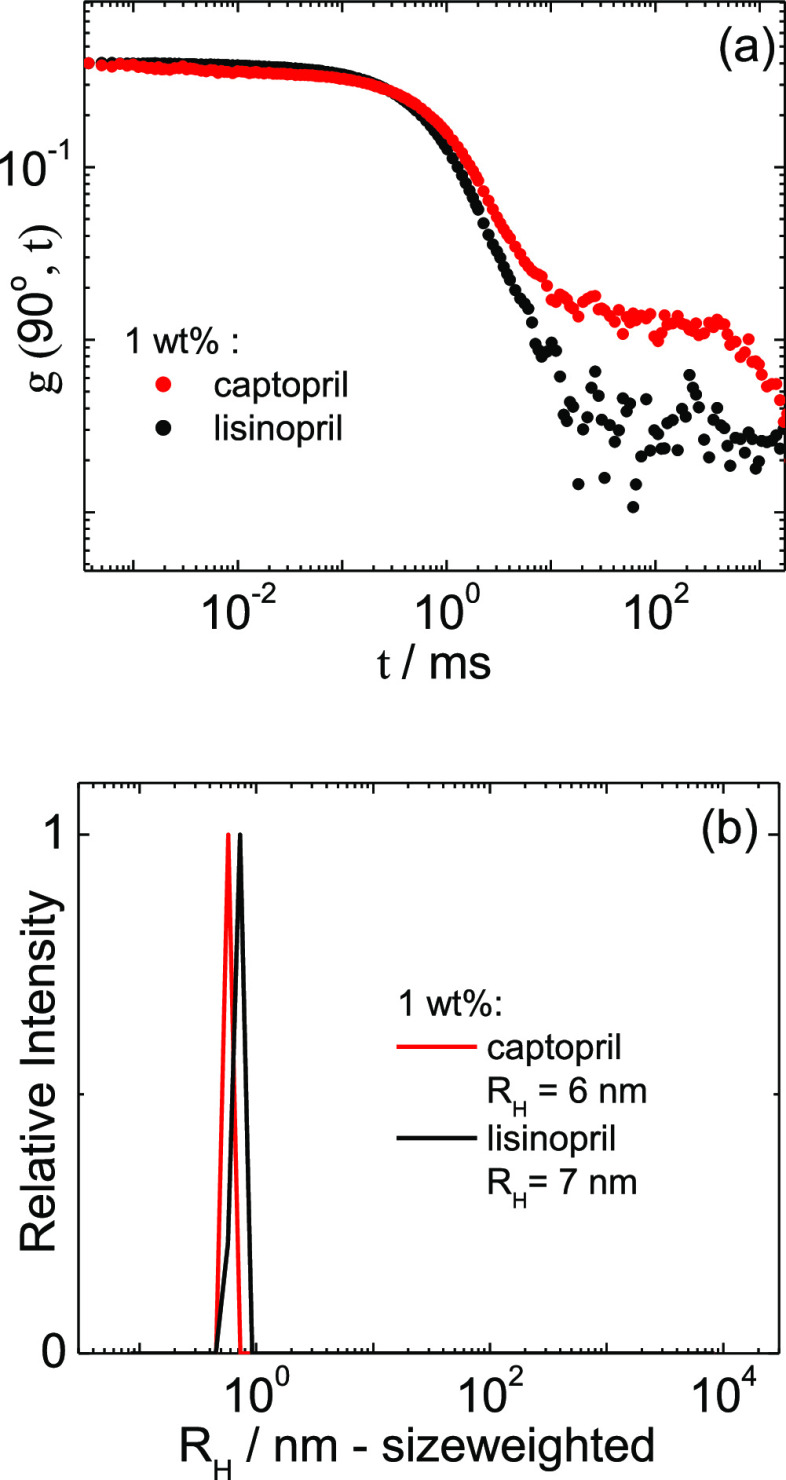
(a) Intensity correlation functions measured for 1 wt % captopril or lisinopril. (b) Number-weighted distribution of hydrodynamic radii calculated from the data in (a).

DLS confirms the presence of the self-assembled aggregates revealed by cryo-TEM images. Figure 7a shows the intensity correlation functions measured for 1 wt % captopril or lisinopril. The size-weighted distribution functions calculated from the data in Figure 7a show the presence of aggregates with a hydrodynamic radius of *R*_H_ = 6 or 7 nm in 1 wt % solutions of captopril or lisinopril, respectively (Figure 7b).

## Conclusions

The combination of surface tensiometry, electrical conductivity, and dye fluorescence measurements provides evidence for the critical aggregation behavior of captopril and lisinopril in water. The consistency between break points in the concentration dependence of these three independent measurements provides high confidence in the existence of a critical aggregation phenomenon in both peptides. Surface tensiometry and electrical conductivity probe distinct types of colligative properties of the molecules, that is, surface assembly and ionic properties in solution, respectively. On the other hand, dye probe fluorescence measurements are sensitive to the formation of local hydrophobic environments. The CAC detected also corresponds to the concentration at which discontinuities are observed in the CD spectra (molar ellipticity at a fixed wavelength corresponding to maxima/minima in the spectra). This suggests that the aggregation may be driven by a molecular conformational switch, which is pH-dependent as suggested by concentration-dependent pH measurements.

The obtained cryo-TEM images and DLS data consistently reveal the presence of small micelle-like aggregates in solution with a diameter <10 nm. The self-assembly of captopril and lisinopril into micelles above a CAC/CMC is not expected based on their molecular structures which do not show typical surfactant characteristics, that is, a lengthy hydrophobic tail group and a hydrophilic head group. However, both molecules bear hydrophobic regions and hydrophilic regions since both molecules have carboxyl units at the C terminus, and lisinopril also bears a cationic lysine residue with the amine unit. We suggest that the hydrophobic interactions between the proline moieties in captopril molecules or the phenylalanine (and/or proline) units in lisinopril molecules respectively balanced by electrostatic interactions may lead to the self-assembly into micelles and that the self-assembly process is driven by a conformational change at the CAC.

We also provide for the first time the SR-XRD crystal structure of captopril and lisinopril. The crystal structures for both agree with previously published laboratory XRD data.

## Supporting Information Available

Electrospray mass spectrometry ionization spectra of captopril and lisinopril, measured pH values for captopril and lisinopril in aqueous solution, FTIR spectrum for captopril, and cryo-TEM images (PDF)

## Notes

The XRD data have been deposited at the CCDC, deposition numbers 2092324 (captopril) and 2092325 (lisinopril).
