# Supplementary material for: Self-Assembly of Angiotensin-Converting Enzyme Inhibitors Captopril and Lisinopril and Their Crystal Structures
Source: Langmuir. 2021 Jul 22;37(30):9170–8. doi: 10.1021/acs.langmuir.1c01340 (PMC8397397; doi:10.1021/acs.langmuir.1c01340)
Supplement: Supplementary file 1 — la1c01340_si_001.pdf [file la1c01340_si_001.pdf]

Supplementary Information

Self-assembly of Angiotensin-Converting Enzyme Inhibitors Captopril and Lisinopril and Their Crystal Structures

Valeria Castelletto,[†,*] Jani Seitsonen,[#] Janne Ruokolainen,[#] Sarah A. Barnett,[×] Callum Sandu[†] and Ian W. Hamley[†,*]

[†] Department of Chemistry, University of Reading, Reading RG6 6AD, United Kingdom.

[#] Nanomicroscopy Center, Aalto University, Puumiehenkuja 2, FIN-02150 Espoo, Finland.

[×] Diamond Light Source, Harwell Science and Innovation Campus, Fermi Avenue, Didcot OX11 0DE, United Kingdom

* Authors for correspondence. v.castelletto@reading.ac.uk; i.w.hamley@reading.ac.uk

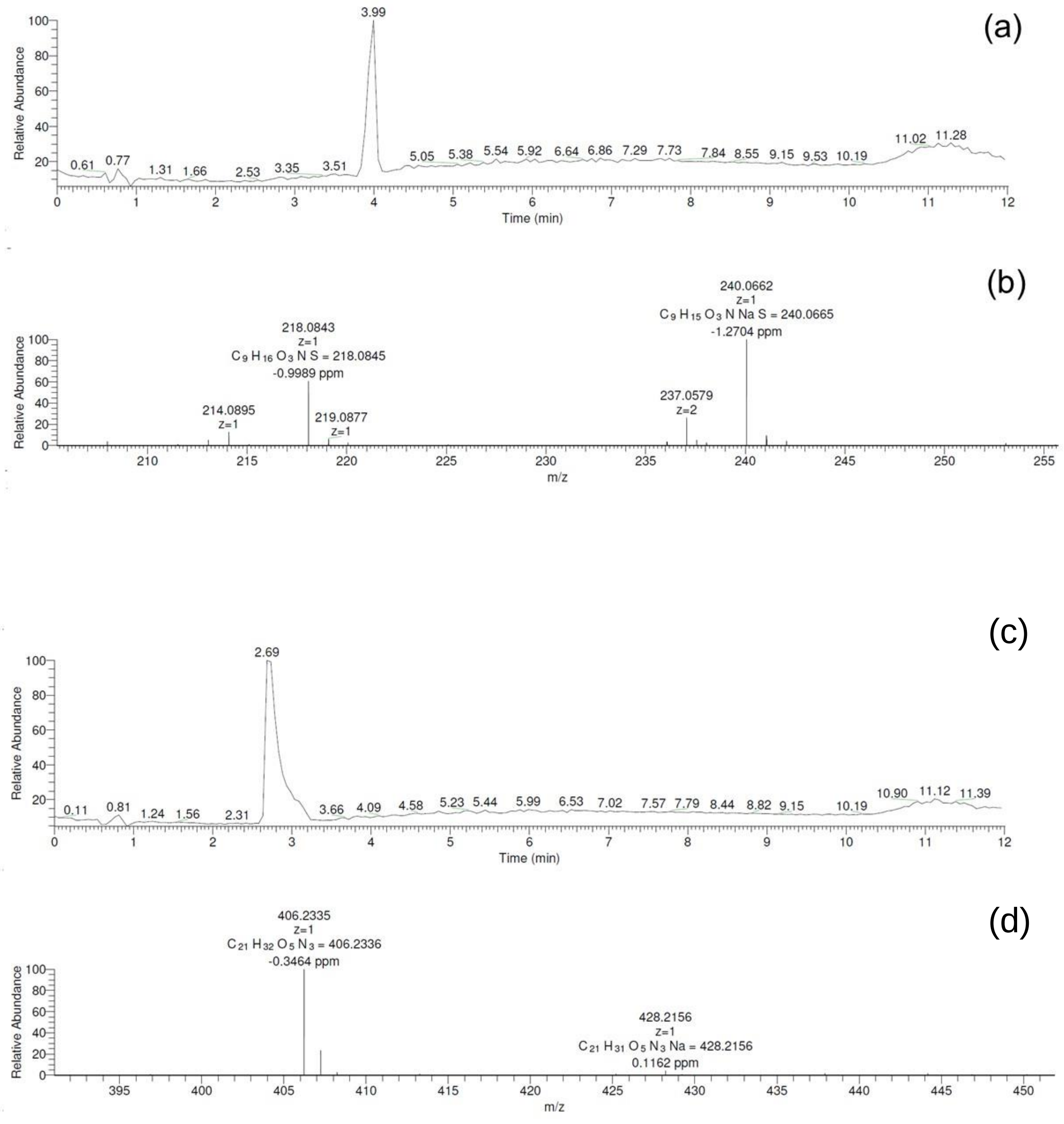

Figure S1. Mass spectra for (a, b) captopril and (c, d) lisinopril.

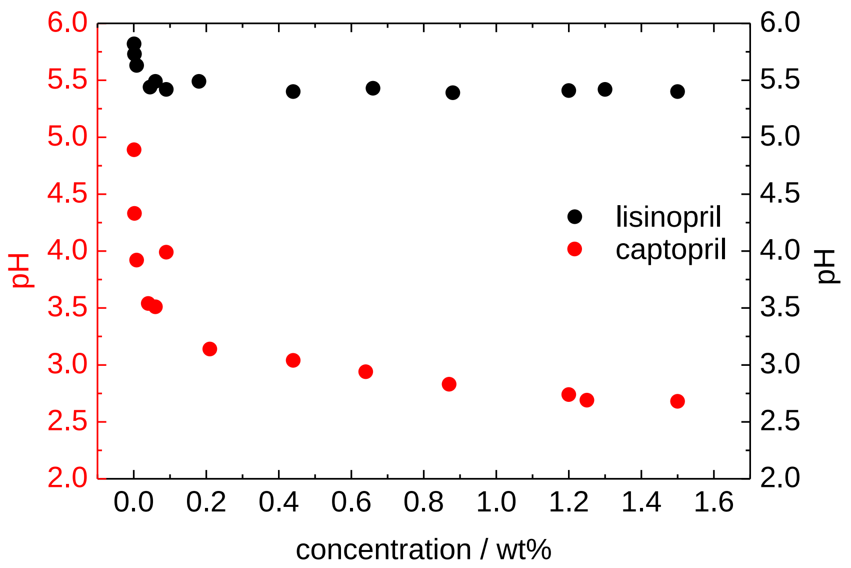

Figure S2. Measured pH values for captopril and lisinopril in aqueous solution.

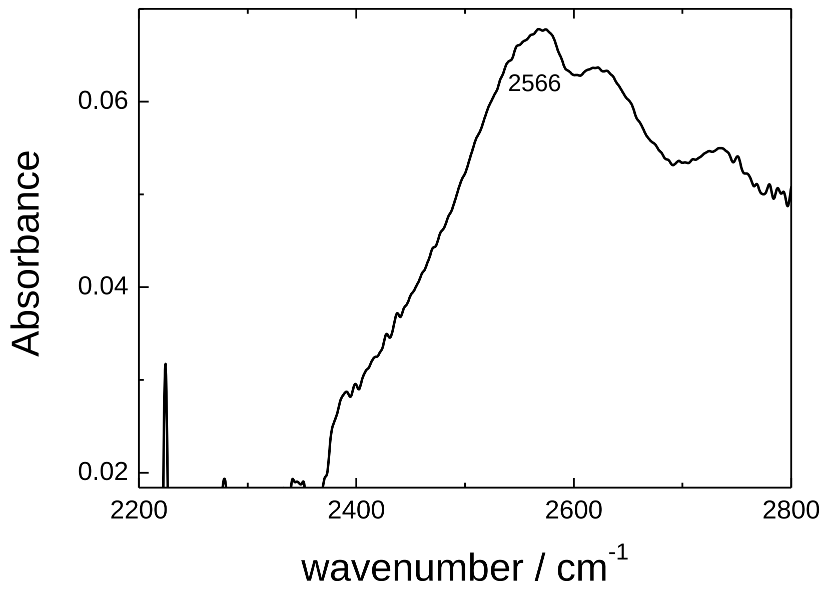

Figure S3. FTIR spectrum for a 1 wt% solution of captopril in H_2O.

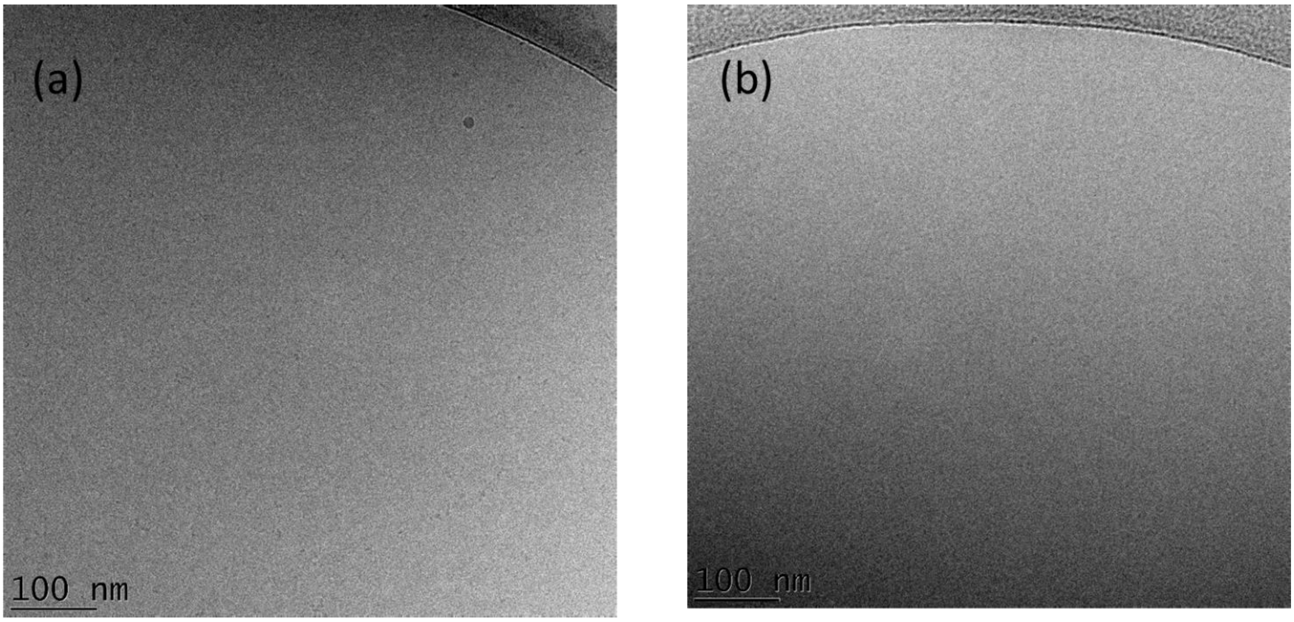

Figure S4. Cryo-TEM images for aqueous solutions of (a) 0.05 wt% captopril, (b) 0.06 wt% lisinopril.
